# Comparative frequency of four different types of pregnancy-associated thyrotoxicosis in a single thyroid centre

**DOI:** 10.1186/s13044-017-0039-0

**Published:** 2017-08-08

**Authors:** Akane Ide, Nobuyuki Amino, Takumi Kudo, Waka Yoshioka, Mako Hisakado, Eijun Nishihara, Mitsuru Ito, Shuji Fukata, Hirotoshi Nakamura, Akira Miyauchi

**Affiliations:** Kuma Hospital, Centre for Excellence in Thyroid Care, 8-2-35 Shimoyamate-dori, Chuo-ku, Kobe, 650-0011 Japan

**Keywords:** Thyrotoxicosis, Pregnancy, Postpartum, Graves’ disease, Destructive thyroiditis

## Abstract

**Background:**

Pregnancy and delivery markedly influence thyroid function. However, the comparative prevalence of gestational thyrotoxicosis (GT), new onset of Graves’ disease during pregnancy (GD during pregnancy), postpartum destructive thyrotoxicosis (PPT), and postpartum Graves’ thyrotoxicosis (PPGD) has not yet been determined.

**Methods:**

We prospectively registered and performed a review of 4127 consecutive non treated female patients with thyrotoxicosis, seen between August 2008 and December 2013 in our outpatient clinic of Kuma Hospital. 187 out of the 4127 women had new diagnosis of thyrotoxicosis during pregnancy or in the postpartum period. We investigated the prevalence of new diagnosis of GT, GD during pregnancy, PPT and PPGD and compared the characteristics of these types of thyrotoxicosis. The postpartum period is defined as twelve months after delivery.

**Results:**

Out of 187 pregnant or postpartum women, we identified 30 (16.0%) with GT, 13 (7.0%) with GD during pregnancy, 42 (22.5%) with PPT, and 102 (54.5%) with PPGD. The onset time of thyrotoxicosis during pregnancy, i.e., both GT and GD during pregnancy, was delayed by a couple of weeks when hCG peaked at 10 gestational weeks. Seventy-six percent of patients with PPT developed thyrotoxicosis between delivery and 4 months postpartum; on the other hand, 83.3% of patients with PPGD developed thyrotoxicosis at 6 months postpartum or later.

**Conclusions:**

We named gestational thyrotoxicosis, new onset of Graves’ disease during pregnancy, postpartum destructive thyrotoxicosis, and postpartum Graves’ thyrotoxicosis as pregnancy-associated thyrotoxicosis. A clinically significant number of women developed Graves’ disease in the postpartum period in a single thyroid centre.

## Background

It is well known that the clinical course of autoimmune thyroid disease is markedly influenced by pregnancy [1]. In early pregnancy, the thyroid is physiologically activated by asialo-hCG [2], and 3.3% of normal Japanese pregnant women showed gestational thyrotoxicosis [3]. After delivery, 5.5% of postpartum women in the general population develop various types of thyroid dysfunction [4]. Korelitz et al. [5] examined the prevalence of thyrotoxicosis among pregnant women in the United States and found 5.9 cases per 1000 pregnant per year. The incidence of PPT was reported between 1.1% and 18.2% [6]. The prevalence of postpartum thyrotoxicosis has been inconsistent in previous studies due to various differences in study and geography.

However, previous data are the results of “screening studies”, and it is unknown whether postpartum thyroiditis is more frequent than postpartum onset of Graves’ disease in “clinical practice”. Therefore, we carefully studied the new onset of thyrotoxicosis during and after pregnancy in our outpatient clinic. These types of thyrotoxicosis were tentatively referred to as pregnancy-associated thyrotoxicosis, and their nature and comparative prevalence were examined. This is the first report on the comparative study of four different types of pregnancy-associated thyrotoxicosis in clinical practice in a single thyroid centre in iodine-sufficient regions in Japan.

## Methods

### Subjects

We performed a retrospective review of 4127 consecutive non treated female patients with thyrotoxicosis seen at Kuma Hospital between August 2008 and December 2013. Of those, 2267 (54.9%) were 20–45 years old. In the present study, childbearing age was defined as between 20 and 45 years of age. The diagnosis of thyrotoxicosis was based on thyroid function tests: suppressed serum TSH and high free T4 (FT4) and T3 (FT3). Patients were followed-up once every two months until they entered euthyroid states or close to the condition. Blood samples were taken during each visit to our clinic. There are four different types of thyrotoxicosis during pregnancy and postpartum: 1) gestational thyrotoxicosis (GT), 2) new onset of Graves’ disease during pregnancy (GD during pregnancy), 3) postpartum destructive thyrotoxicosis (PPT), and 4) postpartum Graves’ thyrotoxicosis (PPGD). In terms of onset, physicians are often not able to detect when hyperthyroidism occurs for the first time because thyrotoxicosis may occur repeatedly. We defined a new onset of Graves’ disease as when patients had never had symptoms of thyrotoxicosis or visited the clinic until they were newly diagnosed with thyrotoxicosis by their obstetrician or by our team. Although we did not perform a radioactive uptake during pregnancy or the postpartum period, we carefully followed all patients with Graves’ disease for several years. We diagnosed cases of GT as patients who had transient thyrotoxicosis without TRAb (TRAb≧2.0 IU/L) or TgAb/TPOAb positivity (TgAb≧40 U/mL/TPOAb≧28.0 U/mL). Cases of Graves’ disease were defined as patients with TRAb positivity. We diagnosed cases of PPT as, patients who had transient thyrotoxicosis postpartum periods without TRAb positivity. The postpartum period is defined as twelve months after delivery. The supplemental ultrasound of thyroid volume and blood flow (TBF) were measured for differential diagnosis. We excluded patients with normal thyroxinemia and suppressed TSH, multinodular toxic goiter, or previous thyroid disease because the aim of the present study was to detect the new onset of thyrotoxicosis during pregnancy and the postpartum period. We defined these types of thyrotoxicosis as pregnancy-associated thyrotoxicosis. The present study was approved by the Institutional Review Board of Kuma Hospital and the Ethics Committee in Kuma Hospital.

### Thyroid function and autoantibodies

TSH, FT4, and FT3 concentrations were measured using chemiluminescent immunoassays (Architect TSH, Architect FT4, and Architect FT3, respectively; Abbott Japan Co., Tokyo, Japan; normal ranges: for 0.3–5.0 μIU/mL for TSH, 0.7–1.6 ng/dL for FT4, and 1.70–3.70 pg/mL for FT3). Serum levels of anti-thyroglobulin antibody (TgAb), anti-thyroid peroxidase antibody (TPOAb), and anti-TSH receptor antibody (TRAb) were measured using electrochemiluminescence immunoassays (ECLusys 2010; Roche Diagnostics Japan Co., Tokyo, Japan; normal range: < 39.9 U/mL for TgAb, < 27.9 U/mL for TPOAb, and <1.9 IU/L for TRAb), as previously reported [7].

### Ultrasound measurements of thyroid volume and blood flow

The diagnostic equipment used were SSA-770A (Aplio 80) and SSA-700A (Aplio 50) ultrasound machines (Toshiba Medical Systems, Otawara, Tochigi, Japan) connected to an 8 MHz linear transducer (PLT-805AT). Thyroid volume and thyroid blood flow (TBF) were measured quantitatively, as reported previously [8]. The quantitative value of thyroid blood flow was expressed as a percent TBF measurement and was reproducible; and intra-assay CV was 4.74% at 22.5% of TBF. The calculation of TBF was so simple that operator difference was negligible. The operators were blinded to the results of the laboratory tests of patients when they performed the ultrasound scans.

### Statistical analysis

Comparisons of various parameters between GT and GD during pregnancy, or PPT and PPGD, were performed by the Mann-Whitney test with StatFlex (version 6.0, Artech Co., Ltd., Osaka). Differences were considered significant at *P* < 0.05.

## Results

There were 4127 new female cases of thyrotoxicosis. Among these, we were able to classify patients with Graves’ disease (*n* = 3494, 84.7%), painless thyroiditis (*n* = 288, 7.0%) subacute thyroiditis (*n* = 297, 7.2%), Plummer’s disease (*n* = 18, 0.4%), and gestational thyrotoxicosis (*n* = 30, 0.7%). Of the 4127 female patients with thyrotoxicosis, 2267 (54.9%) were 20–45 years old. We identified 187 women of childbearing age with pregnancy-associated thyrotoxicosis. Patients with pregnancy-associated thyrotoxicosis consisted of 30 GT (16.0%), 13 GD during pregnancy (7.0%), 42 PPT (22.5%), and 102 PPGD (54.5%).

### Gestational thyrotoxicosis (GT) and new onset of Graves’ disease during pregnancy (GD during pregnancy)

**Fig. 1:**
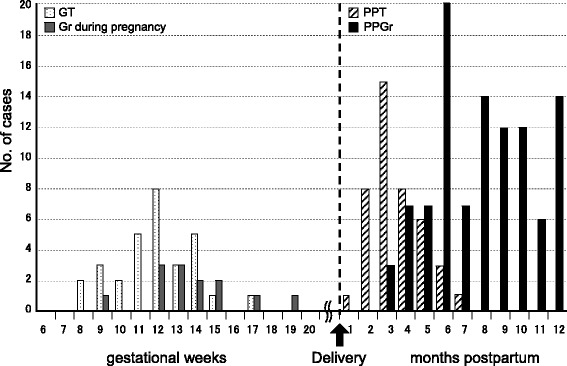
Four different types of pregnancy-associated thyrotoxicosis

Table 1 shows clinical and laboratory data at the onset of thyrotoxicosis among patients with GT and GD during pregnancy. No significant difference was observed in the age at onset between GT and GD during pregnancy; however, there was a significant difference of onset time of thyrotoxicosis between the two (Table 1). The median onset time of GT and GD during pregnancy was 12 and 13 gestational weeks, respectively, and delayed from 10 weeks of hCG peak (Fig. 1). FT4, FT3, and FT3/FT4 were significantly higher in patients with GD during pregnancy than in those with GT. TRAb and thyroid volumes on ultrasonography were significantly higher in patients with GD during pregnancy than in those with GT (*p* < 0.001, Table 1). Eight (61.5%) of 13 patients with GD during pregnancy had a TBF greater than 4.0%; on the other hand, 5 (13.7%) of 30 GT had a TBF greater than 4.0%.

**Table 1:** The median (5th–95th percentile range) values of clinical and laboratory parameters in patients with newly onset of thyrotoxicosis during pregnancy

	Gestational thyrotoxicosis *n* = 30	New onset Graves’ thyrotoxicosis during pregnancy *n* = 13	*p* value**
Onset age (years)	31.0 (24–40)	33.0 (25–37)	0.3257
Onset time (gestational weeks)	12.0 (8–15)	13.0 (10.8–17.8)	0.018
FT4 (ng/dl)	1.96 (1.61–2.52)	2.47 (1.67–3.94)	0.003
FT3 (pg/ml)	4.67 (3.76–6.67)	10.31 (4.72–30.00)	<0.001
FT3/FT4	2.38 (1.98–3.06)	3.66 (2.40–7.47)	<0.001
Thyroid volume (g)	17.1 (11.1–29.8)	27.4 (17.8–40.8)	<0.001
Blood flow (%)	1.6 (0.8–6.6)	6.5 (1.4–18.8)	0.004
TRAb (IU/l)	1.3 (1.3–1.3)*	5.6 (5.6–32.0)	<0.001
TgAb (IU/ml)	28.0 (28.0–42.9)*	28 (28–454.2)*	0.003
TPOAb (IU/ml)	16.0 (16.0–16.8)*	19.3 (16–441.6)*	<0.01
Positive rate of TRAb (%)	0	100	<0.001
Positive rate of TgAb (%)	10.0	46.2	0.007
Positive rate of TPOAb (%)	3.3	46.2	<0.001

*Undetectable levels of TRAb, TgAb and TPOAb less than 1.3 IU/l, 28.0 U/ml and 16.0 U/ml were respectively calculated as 1.3, 28.0 and 16.0. **Comparisons of various parameters between GT and Gr during pregnancy were performed by the Mann-Whitney U-test

All patients with GT improved thyrotoxicosis in the second trimester. They never had positive TRAb and TgAb/TPOAb during pregnancy. In the present study, patients with GD during pregnancy had no history of thyroid disease, nor had they undergone thyroid function testing before they became pregnant. Only one patient had a history of smoking, and four patients had immediate family members with autoimmune thyroid diseases.

Patients with GD during pregnancy had low TRAb levels (median TRAb 5.6 IU/L, 95% range 2.2–32.0 IU/L, Table 1) and 5 (38.5%) of 13 patients with GD during pregnancy had a TBF less than 4.0%. Those with a TBF less than 4.0% had positive TRAb of less than 10.0 IU/L (normal range: <1.9 IU/L for TRAb). Eleven (84.6%) out of 13 patients with GD during pregnancy were administered ATD or KI. Of the four patients who were prescribed KI instead of ATD, three had to be switched from KI to ATD in late pregnancy or after delivery, but the fourth patient was transferred to a different hospital in the second trimester due to severe thyrotoxicosis. Only two patients with GD during pregnancy had course observation. They had mild thyrotoxicosis, low TRAb (2.7, 3.9 IU/L), low TBF (1.8, 2.5%) and mild goiter (21.9, 21.2 g). They delivered without medications or complications. However, they had aggravated thyrotoxicosis with higher TRAb (5.0, 13.7 IU/L) at 4 or 7 months after delivery, and both had to take propylthiouracil. After the initiation of ATD following the diagnosis of GD during pregnancy, most patients stopped taking the medication in late pregnancy because they were in remission. After delivery, patients with GD during pregnancy developed aggravated thyrotoxicosis and were prescribed ATD. We could not make a differential diagnosis from GT or painless thyroiditis.

### Postpartum destructive thyrotoxicosis (PPT) and postpartum Graves’ thyrotoxicosis (PPGD)

Among the 187 cases of pregnancy-associated thyrotoxicosis, the prevalence of postpartum Graves’ disease was the highest (54.5%). The median age at onset was approximately 31 years in patients with PPT or PPGD (Table 2). The onset time of thyrotoxicosis ranged from 1 to 12 months postpartum (Fig. 1). Eighty-five patients (83.3%) who developed thyrotoxicosis at six months or later had PPGD. Thirty-two out of 42 patients (76.2%) who developed thyrotoxicosis between delivery and 4 months postpartum had PPT. Eighty-one (88.0%) out of 92 PPGD patients who underwent the TBF exam had a high TBF of greater than 4.0%, while only one PPT patient had a TBF greater than 4.0%. Serum FT4 and FT3 were significantly higher in patients with PPGD, and the serum FT3/FT4 ratio was also significantly higher in PPGD than in PPT (Table 2). No significant difference was observed in TgAb between patients with PPGD and PPT, but TPOAb in PPGD was significantly higher than that in PPT (Table 2).

**Table 2:** The median (5th–95th percentile range) values of clinical and laboratory parameters in patients with newly onset of thyrotoxicosis after delivery

	Postpartum destructive thyrotoxicosis *n* = 42	Postpartum Graves’ thyrotoxicosis *n* = 102	*p* value**
Onset age (years)	30.0 (22.1–38.0)	31.5 (23–39)	0.5416
Onset time after delivery (months)	3.0 (2.0–6.0)	8.0 (4.0–12.0)	<0.001
FT4 (ng/dl)	2.20 (1.60–4.23)	2.88 (1.84–4.84)	<0.001
FT3 (pg/ml)	7.77 (4.79–30.0)	21.3 (6.62–30.0)	<0.001
FT3/FT4	3.56 (2.56–7.01)	6.25 (6.25–10.36)	<0.001
Thyroid volume (g)	22.8 (11.0–43.9)	25.4 (14.8–57.8)	0.2433
Blood flow (%)	1.4 (0.1–3.7)	8.5 (2.6–24.4)	<0.001
TRAb (IU/l)	1.3 (1.3–1.4)*	10.8 (2.7–116.6)	<0.001
TgAb (IU/ml)	337.6 (28.0–2176.6)*	267.9 (28.0–1105.3)*	0.4135
TPOAb (IU/ml)	16.0 (16.0–600.0)*	87.0 (16.0–600.0)*	0.026
Positive rate of TRAb (%)	0	100	<0.001
Positive rate of TgAb (%)	88.1	76.5	0.1139
Positive rate of TPOAb (%)	45.1	64.7	0.031

*Undetectable levels of TRAb, TgAb and TPOAb less than 1.3 IU/l, 28.0 U/ml and 16.0 U/ml were respectively calculated as 1.3, 28.0 and 16.0. **Comparisons of various parameters between PPGr and PPT were performed by the Mann-Whitney U-test

## Discussion

It is well known that Graves’ disease deteriorates in early pregnancy. HCG has been shown to exhibit immunoregulatory properties, i.e., it suppresses the mitogen-induced responses of T and B cells by inducing suppressor T cells in order to contribute to maternal tolerance towards the foetus [9–12]. In early pregnancy, a large amount of hCG has been suggested to bind to the TSH receptor [13, 14] and stimulate thyroid function in vitro [14] and in vivo [2]. Gestational thyrotoxicosis is known to be caused by hCG in early pregnancy [15].

The mechanism underlying the onset of Graves’ disease in early pregnancy may be partly explained by the role of hCG. Mild aggravation of thyrotoxicosis in early pregnancy due to hCG stimulation has been reported in Graves’ patients who were in a state of near remission [16]. An increase in serum-thyroxine binding globulin (TBG) concentrations and stimulation of the TSH receptor by hCG greatly affect thyroid function during pregnancy. Serum TBG concentrations have been shown to increase by almost two-fold by the effects of oestrogen due to the decreased clearance of TBG [17]. Therefore, thyroxine and triiodothyronine production increase during the first half of pregnancy. An upsurge occurs in serum hCG soon after fertilization and peaks at 10 to 12 gestational weeks. TSH and hCG are members of a glycoprotein hormone family that contains common α-subunits, and the TSH receptor is stimulated by asialo-hCG [2]. A previous study found no increase in TRAb levels in early pregnancy among women with known Graves’ disease who were in remission before pregnancy [16, 18]. Maternal immune activities are generally suppressed in order to prevent rejection of the developing foetus during pregnancy. During pregnancy, the symptoms of Graves’ disease have been reported to remain unchanged or become ameliorated but are rarely aggravated. The prevalence of hyperthyroidism due to Graves’ disease in pregnancy was previously reported to be approximately 0.1–0.4% of patients [18, 19]. The mechanism of new onset of Graves’ disease during pregnancy currently remains unclear; however, many immunological mechanisms likely contribute to the mother’s autoimmunity.

In the present study, all patients with GD during pregnancy were TRAb-positive, whereas none of patients with GT were. TBF may not be a sensitive test for making a differential diagnosis in early pregnancy. TRAb is considered to be the most sensitive and specific test for differentiating not only postpartum but also pregnancy-associated thyrotoxicosis [7]. A final diagnosis should be confirmed by the follow-up of these patients, and in some cases, it still may not be possible to make a differential diagnosis.

Previous studies reported that the postpartum aggravation of autoimmune thyroid diseases through the immune rebound mechanism may lead to thyrotoxicosis [20, 21]. The postpartum rebound of immune activities consists of 2 phases. Cellular immunity becomes active from 1 to 4 months postpartum, whereas the activity of humoural immunity increases from 7 to 10 months postpartum [22, 23]. We previously reported the usefulness of TRAb and TBF to differentiate postpartum thyrotoxicosis [7]. In this study, PPT occurs from 1 to 7 months postpartum, whereas Graves’ disease develops from 3 to 12 months postpartum. Patients with Graves’ disease commonly relapse during the postpartum period, even if they were in remission before pregnancy [21, 24, 25]. In the present study, patients with PPGD had no history of thyroid disease, goiter, or thyrotoxicosis. Eighty-one (88.0%) of 92 patients with PPGD had TBF greater than 4.0%; on the other hand, 11 (12.0%) of patients with PPGD had TBF less than 4.0%, even though they had thyrotoxicosis and TRAb positivity. However, observing TRAb positivity and detecting TBF levels greater than 4.0% using ultrasound were useful for diagnosing PPGD. Increase of anti-thyroid antibody titers peaks at around 6 months postpartum [26]. Therefore titers of TPOAb are higher at 8 months postpartum in Graves’ disease than 3 months postpartum in postpartum thyroiditis. Titers of TgAb in autoimmune (Hashimoto’s) thyroiditis are usually higher than that of Graves’ disease, possibly due to the increase of circulating thyroglobulin concentration in Graves’ disease. These possibly explain the no significant difference of TgAb titers between PPT and PPGD.

Previous studies proposed that postpartum thyroiditis was one of the most common endocrine disorders, including subclinical hyperthyroidism [4, 27–29]. Andersen et al. reported a population-based study of the incidence of maternal hyperthyroidism in Denmark [30]. They concluded that a risk of onset of Graves’ disease was associated with both early pregnancy and the postpartum period [30]. In this study, the prevalence of patients with PPGD was more than twice that of patients with PPT in our single hospital. Several factors may explain these results. We excluded patients with mild thyroid dysfunction, i.e., normal FT4 and suppressed TSH (less than 0.3); there were 42 patients with subclinical hyperthyroidism. These patients were assumed to recovery in a relatively short period after destructive thyrotoxicosis. Furthermore, since this transient thyroiditis most often occurs in the very early postpartum period [31], patients with PPT had milder symptoms and improved more quickly, and they were less likely to visit our clinic. PPT has a typical pattern of transient hyperthyroidism followed by hypothyroidism before returning to a euthyroid state within 6 months [32]. On the other hand, the symptoms of thyrotoxicosis become exacerbated in patients with PPGD over time, and then they visit a clinic.

Our study had some limitations. First, the design was retrospective, and second, the onset of thyrotoxicosis might not be clear because the patients who were referred by another clinic visited our clinic several weeks later. Third, the patients did not have radioactive iodine uptake tests because they were pregnant or breast-feeding, which can lead to uncertainty in making differential diagnosis.

## Conclusion

In this study, we classified pregnancy-associated thyrotoxicosis into four different types in a single hospital for the first time. GT, GD during pregnancy, PPT, and PPGD were detected in 16.0%, 7.0%, 22.5%, and 54.5%, respectively, of 187 female patients of child-bearing age. A clinically significant number of women developed Graves’ disease in the postpartum period, and the prevalence of new onset of Graves’ disease during pregnancy was less than that of gestational thyrotoxicosis and postpartum Graves’ disease.

## References

[1] Amino N, Tada H, Hidaka Y (1996). Autoimmune thyroid disease and pregnancy. J Endocrinol Investig.

[2] Tsuruta E, Tada H, Tamaki H, Kashiwai T, Asahi K (1995). Pathogenic role of asialo human chorionic gonadotropin in gestational thyrotoxicosis. J Clin Endocrinol Metab.

[3] Orito Y, Oku H, Kubota S, Amino N, Shimogaki K (2009). Thyroid function in early pregnancy in Japanese healthy women: relation to urinary iodine excretion, emesis, and fetal and child development. J Clin Endocrinol Metab.

[4] Amino N, Mori H, Iwatani Y, Tanizawa O, Kawashima M (1982). High prevalence of transient post-partum thyrotoxicosis and hypothyroidism. N Engl J Med.

[5] Korelitz JJ, McNally DL, Masters MN, Li SX, Xu Y, Rivkees SA (2013). Prevalence of thyrotoxicosis, antithyroid medication use, and complications among pregnant women in the United States. Thyroid.

[6] Stangnaro-Green A (2012). Approach to the patient with postpartum thyroiditis. J Clin Endocrinol Metab.

[7] Ide A, Amino N, Kang S, Yoshioka W, Nakamura H (2014). Differentiation of postpartum Graves’ thyrotoxicosis from postpartum destructive thyrotoxicosis using antithyrotropin receptor antibodies and thyroid blood flow. Thyroid.

[8] Ota H, Amino N, Morita S, Kobayashi K, Kubota S, et al. Quantitative measurement of thyroid blood flow for differentiation of painless thyroiditis from Graves’ disease. Clin Endocrinol(Oxf). 2007;67:41-45. 10.1111/j.1365-2265.2007.02832.x 17437515

[9] Adcock EW, Teasdale T, August CS, Cox S, Meschia G (1973). Human chorionic gonadotropin: its possible role in maternal lymphocyte suppression. Science.

[10] Fuchs T, Hammarstrom L, Smith CI, Brundin J (1982). Sex-dependent induction of human suppressor T cells by chorionic gonadotropin. J Reprod Immunol.

[11] Davies TF, Taliadouros GS, Catt KJ, Nisula BC (1979). Assessment of urinary thyrotropin-competing activity in choriocarcinoma and thyroid disease: further evidence for human chorionic gonadotropin interacting at the thyroid cell membrane. J Clin Endocrinol Metab.

[12] Azukizawa M, Kurtzman G, Pekary AE, Hershman JM (1977). Comparison of the binding characteristics of bovine thyrotropin and human chorionic gonadotropin to thyroid plasma membranes. Endocrinology.

[13] Hoermann R, Keutmann HT, Amir SM (1991). Carbohydrate modifications transform human chorionic gonadotropin into a potent stimulator of adenosine 3′,5′-monophosphate and growth responses in FRTL-5 thyroid cells. Endocrinology.

[14] Kimura M, Amino N, Tamaki H, Ito E, Mitsuda N, et al. Gestational thyrotoxicosis and hyperemesis gravidarum: possible role of hCG with higher stimulating activity. Clin Endocrinol(Oxf). 1993;38:345-350. 10.1111/j.1365-2265.1993.tb00512.x 8319364

[15] Tamaki H, Itoh E, Kaneda T, Asahi K, Amino N (1993). Crucial role of serum human chorionic gonadotropin for the aggravation of thyrotoxicosis in early pregnancy in Graves’ disease. Thyroid.

[16] Ain KB, Mori Y, Refetoff S (1987). Reduced clearance rate of thyroxine-binding globulin (TBG) with increased sialylation: a mechanism for estrogen-induced elevation of serum TBG concentration. J Clin Endocrinol Metab.

[17] Tamaki H, Amino N, Aozasa M, Mori M, Tanizawa O (1987). Serial changes in thyroid-stimulating antibody and thyrotropin binding inhibitor immunoglobulin at the time of postpartum occurrence of thyrotoxicosis in Graves’ disease. J Clin Endocrinol Metab.

[18] Mestman JH (2004). Hyperthyroidism in pregnancy. Best Pract Res Clin Endocrinol Metab.

[19] Amino N, Tada H, Hidaka Y, Izumi Y (2000). Postpartum autoimmune thyroid syndrome. Endocr J.

[20] Amino N, Tada H, Hidaka Y (1999). Postpartum autoimmune thyroid syndrome: a model of aggravation of autoimmune disease. Thyroid.

[21] Watanabe M, Iwatani Y, Kaneda T, Hidaka Y, Amino N (1997). Changes in T, B, and NK lymphocyte subsets during and after normal pregnancy. Am J Reprod Immunol.

[22] Shimaoka Y, Hidaka Y, Tada H, Nakamura T, Amino N (2000). Changes in cytokine production during and after normal pregnancy. Am J Reprod Immunol.

[23] Amino N, Tanizawa O, Mori H, Iwatani Y, Yamada T (1982). Aggravation of thyrotoxicosis in early pregnancy and after delivery in Graves’ disease. J Clin Endocrine Metab.

[24] Rotondi M, Cappelli C, Pirali B, Pirola I, Magri F (2008). The effect of pregnancy on subsequent relapse from Graves’ disease after a successful course of antithyroid drug therapy. J Clin Endocrinol Metab.

[25] Freeman R, Rosen H, Thysen B (1986). Incidence of thyroid dysfunction in an unselected postpartum population. Arch Intern Med.

[26] Amino N, Kuro R, Tanizawa O, Tanaka F, Hayashi C (1978). Changes of serum anti-thyroid antibodies during and after pregnancy in autoimmune thyroid diseases. Clin Exp Immunol.

[27] Fung HY, Kologlu M, Collison K, John R, Richards CJ (1988). Postpartum thyroid dysfunction in mid Glamorgan. Br Med J (Clin Res Ed).

[28] Gerstein HC (1990). How common is postpartum thyroiditis? A methodologic overview of the literature. Arch Intern Med.

[29] Vargas MT, Briones-Urbina R, Gladman D, Papsin FR, Walfish PG (1988). Antithyroid microsomal autoantibodies and HLA-DR5 are associated with postpartum thyroid dysfunction: evidence supporting an autoimmune pathogenesis. J Clin Endocrinol Metab.

[30] Anderson SL, Olsen L, Carle A, Laurberg P (2015). Hyperthyroidism incidence fluctuates widely in and around pregnancy and is at variance with some other autoimmune disease: a Danish population-based study. J Clin Endocrinol Metab.

[31] Nohr SB, Jorgensen A, Pedersen KM, Laurberg P (2000). Postpartum thyroid dysfunction in pregnant thyroid peroxidase antibody-positive women living in an area with mild to moderate iodine deficiency: is iodine supplementation safe?. J Clin Endocrinol Metab.

[32] Amino N, Tada H, Hidaka Y (1996). The spectrum of postpartum thyroid dysfunction: diagnosis, management, and long-term prognosis. Endocr Prac.

